# Determinants of excellent/good self-rated health among HIV positive individuals in South Africa: evidence from a 2012 nationally representative household survey

**DOI:** 10.1186/s12889-018-5102-9

**Published:** 2018-01-30

**Authors:** M. L. H. Mabaso, N. P. Zungu, T. Rehle, S. Moyo, S. Jooste, K. Zuma

**Affiliations:** 1 Epidemiology and Strategic Information Unit, HIV/AIDS, STIs and TB Programme, Human Sciences Research Council, Private Bag X07, Dalbridge, Durban, 4014 South Africa; 2 HIV/AIDS, STIs and TB, Human Sciences Research Council, Pretoria, South Africa; 3 School of Public Health and Family Medicine, University of Cape Town, Cape Town, South Africa; 4 HIV/AIDS, STIs and TB, Human Sciences Research Council, Cape Town, South Africa; 5 Research Methodology and Data Center, Human Sciences Research Council, Pretoria, South Africa

**Keywords:** Determinants, Self-rated health, Excellent/good health, HIV, South Africa

## Abstract

**Background:**

In South Africa, HIV is increasingly becoming a chronic disease as a result of advances in HIV treatment and prevention in the last three decades. This has changed the perception from a life threating to a potentially manageable disease. However, little is known about self-perceived health status of HIV-infected individuals. Self-rated health (SRH) has been shown to be a sensitive indicator of health-relatedchanges directly linked to HIV, but can also be influenced by differences in social and material conditions. The aim of this paper was to identify determinants of excellent/good SRH among HIV-infected individuals using socio-demographic, life style and health related data.

**Methods:**

The study used data from the nationally representative 2012 South African population-based household survey on HIV prevalence, incidence and behaviour conducted using multi-stage stratified cluster sampling design. Bivariate and multivariate logistic regression models were used to identify determinants of SRH among HIV-infected individuals.

**Results:**

Out of a total of 2632 HIV positive participants 74.1% (95% CI: 68.4-74.2) reported excellent/good SRH. Increased likelihood of reporting excellent/good SRH was significantly associated with being Black African [OR= 1.97 (95%CI: 1.12-3.46), *p* = 0.019] and belonging to least poor household [OR= 3.13 (95%CI: 1.26-7.78), *p* = 0.014]. Decreased likelihood of reporting excellent/good SRH was significantly associated with those aged 25 to 34 years [OR= 0.49 (95% CI: 0.31-0.78), *p* = 0.003], 35 to 44 years[OR= 0.27 (95% CI: 0.17-0.44), *p* < 0.001], 45 to 54 years [OR= 0.20 (95% CI: 0.12-0.34), *p* < 0.001], and those 55 years and older [OR= 0.15 (95% CI: 0.09-0.26), *p* < 0.001], hospitalization in the past twelve months [OR= 0.40 (95% CI: 0.26-0.60), *p* < 0.001].

**Conclusion:**

To have positive health effects and improve the perceived health status for PLWH social interventions should seek to enhance to support for the elderly HIV-positive individuals, and address the challenge of socio-economic inequalities and underlying comorbid conditions resulting in hospitalization.

## Background

South Africa has the biggest HIV epidemic in the world, and in 2012 an estimated 6.4 million people were living with HIV [1]. The epidemic has had a negative impact on the health, social, economic and demographic aspects of life in the country [2]. However, expansion of HIV testing, treatment and prevention programmes have resulted in significant clinical improvement and reduction in HIV related hospitalization and mortality [3]. Consequently, people living with HIV (PLHIV) have had a greatly improved life expectancy. All these successes including mass media, social and behavioural change communication campaigns against HIV have changed the perception of HIV from a life threating to a potentially manageable disease especially among PLHIV [1, 2, 4].

However, little is known about general health perception and well-being of PLHIV. Self-rated health (SRH) is considered an important indicator for assessing personal perception of health status and satisfaction with life in general in relation to clinical status [5]. SRH is a subjective perception of individual’s sense of well-being, and refers not just to feelings of pain and discomfort, but also to the psychological and social consequences of having a health problem [5]. SRH has been shown to be influenced by differences in social and material conditions attributed to predisposing demographic and socio-economic factors such as age, gender, education level and employment status, place residence/geographic location and behavioural/lifestyle factors such as smoking, alcohol use, and physical inactivity [5–10].

Few available studies among PLHIV have found SRH to be a sensitive indicator of health-related changes directly linked to HIV [5, 11–17]. Some studies found interaction between SRH and AIDS related symptoms, antiretroviral (ARV) treatment and quality of life [12–18]. Others have found an association between SRH and socio-demographic factors such as age, educational level and work situation [11–13, 17], including history of hospitalization [18]. However, these studies have been limited to specific sub-populations and fewer have been conducted in South Africa [14, 16, 17].

In South Africa HIV is increasingly becoming a chronic disease and it is imperative to have a better understanding of factors affecting subsequent change in SRH among PLHIV. The examination of the determinants that have a significant effect on SRH given changes in health status and improved survival is vital for establishing effective strategies prolonging life among PLHIV. The aim of this paper was to examine determinants of excellent/good self-rated health SRH among HIV-infected individuals in South Africa using socio-demographic, life style and health related data from the 2012 nationally representative household survey on HIV prevalence, incidence and behaviour.

## Methods

### Study design and sample

The analysis used data from the 2012 South African HIV prevalence, incidence and behaviour survey, a nationally representative population-based household survey, described in detail elsewhere [1]. Basically, participants were selected using multi-stage stratified cluster sampling using a stratified master sample of 1000 census enumerator areas (EA) sampled from a South African listing of 86,000 EAs from the 2001 population census stratified by province, race group and locality type (urban/rural and formal/informal). In each EA a systematic random sample of 15 households was sampled yielding a total sample size of 15,000 households targeted for the survey.

Persons of all ages living in selected households were all eligible to participate in the study.

Four questionnaires were administered to persons of different age groups in the household each containing various age-specific modules soliciting information related to socio-demographic factors, HIV knowledge, attitudes, practices, and behaviours including general health [1]. All youth and adults who participated provided either written or verbal consent, including parent/guardian informed consent for youth under 18 years of age, and youth verbal assent to have a blood specimen taken.

### HIV testing and estimation of ARV exposure

Dried blood spots (DBS) specimens collected by nurses were tested anonymously for HIV antibodies using a testing algorithm with three different immunoassays (Vironostika HIV Uni-Form II plus O, Biomeriux, Boxtel, The Netherlands; Advia Centaur XP, Siemens Medical Solutions Diagnostics, Tarrytown, NJ, USA; (Roche Elecys 2010 HIV Combi, Roche Diagnostics, Mannheim, Germany). Samples that tested positive for HIV-1 antibodies were tested for the presence of ARVs using high performance liquid chromatography (HPLC) coupled with tandem mass spectrometry. Zidovudine, Nevirapine, Efavirenz, Lopinavir, Atazanavir, and Darunavir were detected using an Applied Biosystems API 4000 tandem mass spectrometer. The limit of detection was set to 0.2 micrograms/ml.

A total of 42,950 individuals in the valid households were eligible to be interviewed, and 38,431 agreed to be interviewed. This paper analysed a subsample of the data collected from persons aged 15 years and older who were HIV positive and who responded to questions on SRH in the health module.

### Self-rated health

The primary outcome variable SRH which was assessed by the question by the question: In general, would you say that your health is excellent, good, fair or poor? The responses were ranked on a 5-point scale from 1 to 4 (excellent = 1; good = 2; fair = 3; bad = 4). For the present analyses, these responses were dichotomised into two categories, good or excellent = 1 and poor or fair = 0.

### Socio-demographic, life style and health-related variables

Socio-demographic characteristics included sex (male and female), age (15–24, 25–34, 35–44, 45–54, 55 years and older), race (Black Africans or other races), marital status (married or not married), educational qualifications (no education/primary, secondary, tertiary) employment status (yes or no), asset based wealth index (low, middle, high) and locality type (urban formal, urban informal, rural informal, and rural formal areas). Including asset based socio-economic status (SES) constructed using multiple correspondence analyses (MCA) based on questions on availability / ownership of broad range of household assets ownership and access to utilities. MCA calculated a composite indicator score computed by adding up all weighted responses [19]. The predicted score for each household was used to compute five quintiles (1st lowest, 2nd lower, 3rd middle, 4th higher and 5th highest) representing a continuum of household SES from the most poor to the least poor.

Life style-related factors comprised of the Alcohol Use Disorder Identification Test (AUDIT) scale (abstainers, low risk drinkers (1–7), high risk drinkers (8–19), hazardous drinkers (20+) [20]. Including questions on recreational drug use (yes or no), and participation in vigorous (yes or no) and moderate sport (yes or no). Health-related factors were based on the following questions: (1) when was the last time you went to see a health personnel (never, within the past 6 months, more than 6 months but not more than a year ago, more than 1 year ago)? (2) Where do you usually obtain health care (Public sector or Private sector)? (3) In the past 12 months, have you been hospitalized for any illness (yes or no)? (4) Self-reported awareness of the HIV status and ARV status based on DBS (not aware of HIV status, aware of HIV status and not on ARVs, aware of HIV status and on ARVs).

### Statistical analysis

All data were analysed using statistical software STATA version 13.0 (Stata Corp, College Station, Texas, USA). The “svy” command was used to introduce weights which take into account the complex design of the survey. Descriptive statistics (frequency distribution and percentages) were used to characterize respondent’s SRH by socio-demographic, life style and health-related factors. The Chi-square test was used compare differences in proportions between categorical variables. Bivariate logistic regression models were used to identify potential factors associated with SRH. Statistically significant variables from the bivariate analysis were entered into a multivariate logistic regression model to examine the independent effects of covariates associated with SRH. Unadjusted odds ratios. (OR), adjusted odds ratio (AOR) and their 95% confidence intervals (CI) with a *p*-value less than 0.05 are reported. Coefficient plots were used to display the results of the final multivariate model [21].

## Results

### Descriptive characteristics

Table 1 shows socio-demographic characteristics of study participants and proportion of reported good/excellent SRH. The majority of study participants were female, 25–34 years old, Black African, unemployed, from poor households and rural informal areas. Out of a total of 2632 HIV positive participants 74.1% (95% CI: 68.4–74.2) reported excellent/good SRH. The proportion of reported good/excellent SRH was significantly higher among those aged 15–24 years, Black Africans, and employed participants.

**Table 1:** Socio-demographic characteristics and reported good/ excellent self-rated health (SRH) among HIV positive participants 15 years and older

Variables	Total		Reported good/excellent SRH			
Sex	n	%	n^a^	%	95% CI	*p*-value*
Male	819	37.1	810	71.0	66.5–76.0	0.824
Female	1813	62.9	1790	71.7	68.5–74.9	
Age in years						
15–24	399	12.5	394	88.4	84.0–91.7	< 0.001
25–34	926	39.4	916	77.0	72.5,80.9	
35–44	693	30.9	686	65.3	59.4,70.8	
45–54	393	12.5	386	59.3	51.2,66.9	
55+	221	4.8	218	52.4	44.1,60.6	
Race groups						
Black African	2398	97.3	2368	71.9	68.9–74.7	0.008
Other	234	2.7	232	56.1	43.3–68.1	
Employment status						
No	1574	63.6	1563	68.9	65.2–72.5	0.029
Yes	916	36.4	907	75.2	70.4–79.5	
Asset based SES^b^						
1st Quintile	923	35.4	917	69.4	64.8–73.6	0.079
2nd Quintile	842	33.3	827	74.3	69.4–78.7	
3rd Quintile	524	22.2	516	72.7	67.2–77.5	
4th Quintile	231	7.6	230	63.5	51.8–73.8	
5th Quintile	83	1.5	83	85.9	73.2–93.2	
Locality Type						
Urban formal	907	37.3	892	71.6	66.1–77.1	0.895
Urban informal	527	12.7	522	69.4	64.4–74.0	
Rural informal	854	44.9	848	71.5	67.4–75.9	
Rural formal	344	5.1	338	74.4	60.8–84.4	

^a^Totals do not add to overall total due to non-response and /or  missing data; ^b^Quintiles 1–5 represent a continuum of household socio-economic status (SES) from the most poor to the least poor; *Significant at *p* < 0.05

Table 2 shows life style and health-related characteristics of study participants and proportions of reported excellent/good SRH. The majority of the study participants were abstainers, did not engage in recreational drug use, did not do moderate and vigorous sport, reported visiting health personnel within the past 6 months, obtained health care from the public sector, were not hospitalized for any illness in the past 12 months, did not report presence of chronic medical conditions and about half were not aware of their HIV status. The proportion of reported excellent/good SRH was significantly higher among participants doing moderate and vigorous intensity sport, those who reported seeing a health personnel more than 6 month and a year ago, those who reported not to have been hospitalized for any illness in the last 12 months, and those aware of their HIV status but not on ARVs.

**Table 2:** Life style and health-related characteristics and reported good/ excellent self-rated health (SRH) among HIV positive participants 15 years and older

Variables	Total		Reported good/excellent SRH			
Alcohol use risk score^b^	n	%	n^a^	%	95% CI	*p*-value*
Abstainers	1662	71.3	1659	73	69.6–76.1	0.744
Low risk drinkers (1–7)	451	19.5	449	71.9	64.9–78.1	
High risk drinkers (8–19)	193	7.5	193	72.3	60.6–81.6	
Hazardous drinkers (20+)	34	1.7	34	60.3	34.1–81.7	
Recreational drug use						
No	2387	95.1	2381	72.2	69.2,74.9	0.321
Yes	123	4.9	123	65.6	50.9–77.8	
Do you do any vigorous intensity sport?						
No	628	24.5	627	76.6	71.1–81.3	0.030
Yes	1971	75.5	1966	69.7	66.2–73.0	
Do you do any moderate intensity sport?						
No	503	20.6	503	79.6	73.3–84.7	0.004
Yes	2097	79.4	2091	69.3	66.0–72.5	
When was the time you went to see a health personnel?						
Within the past six months	1475	56.1	1473	61.6	57.4–65.6	< 0.001
More than six months but not more than a year ago	407	15.5	406	84.7	79.6–88.6	
More than one year ago	601	23.7	600	85.2	81.1–88.6	
Never	110	4.7	109	77.5	58.6–89.3	
Where do you usually obtain health care?						
Public sector	2146	85.6	2141	71.2	68.0,74.2	0.776
Private sector	343	14.4	343	72.5	63.7,79.8	
In the past 12 months, have you been hospitalized for any illness?						
No	258	10	257	54.8	46.7,62.7	< 0.001
Yes	2345	90	2339	73.5	70.4,76.4	
HIV status awareness and treatment						
Not aware of HIV status	1276	50.3	1272	71.9	67.8,75.7	0.009
Aware of HIV status and not on ARVs	824	31.9	821	75.1	70.3–79.4	
Aware of HIV status and on ARVs	463	17.8	462	63.1	56.2–69.5	

^a^Totals do not add to overall total due to non-response and /or missing data; ^b^Risk score based on a questionnaire for Alcohol Use Disorder Identification Test (AUDIT); * Significant at *p* < 0.05

### Bivariate logistic regression analysis

Table 3 shows unadjusted ORs and 95% confidence intervals for bivariate association between reported good/excellent SRH and selected socio-demographic variables. Decreased likelihood of reporting good/excellent SRH was significantly associated increasing age. Increased likelihood of reporting good/excellent SRH was significantly associated with being Black African, employed and living in list poor households. Table 4 shows unadjusted ORs and 95% confidence intervals for bivariate association between reported good/excellent SRH and selected life style and health-related variables. Increased likelihood of reporting good/excellent SRH was significantly associated with doing moderate and vigorous intensity sport. Decreased likelihood of reporting good/excellent SRH was significantly associated with seeing health personnel more than 6 month and a year ago, those who reported not to have been hospitalized for any illness in the last 12 months, and those aware of HIV status and on ARVs.

**Table 3:** Bivariate analysis of socio-demographic factors associated with good/excellent self-rated health among HIV positive participants 15 years and older

	Unadjusted OR	95% CI		*p*-value*
Sex				
Male	Ref			
Female	1.03	0.79	1.35	0.824
Age in years				
15–24	Ref			
25–34	0.44	0.28	0.67	<.0001
35–44	0.25	0.16	0.38	<.0001
45–4	0.19	0.12	0.31	<.0001
55+	0.14	0.09	0.24	<.0001
Race groups				
Black African	Ref			
Other	2.00	1.19	3.35	0.009
Employment status				
No	Ref			
Yes	1.37	1.03	1.82	0.030
Asset based SES^a^				
1st Quintile	Ref			
2nd Quintile	1.28	0.94	1.74	0.119
3rd Quintile	1.17	0.84	1.64	0.352
4th Quintile	0.77	0.46	1.29	0.314
5th Quintile	2.69	1.17	6.17	0.020
Locality Type				
Urban formal	Ref			
Urban informal	0.90	0.63	1.28	0.554
Rural informal	0.99	0.71	1.40	0.970
Rural formal	1.15	0.57	2.31	0.697

*OR* Odds ratio; *CI* confidence interval; ^a^Quintiles 1–5 represent a continuum of household socio-economic status (SES) from the most poor to the least poor; *Significant at *p* < 0.05

**Table 4:** Bivariate analysis of Life style and health-related factors associated with good/excellent self-rated health among HIV positive participants 15 years and older

Alcohol use risk score^a^	Unadjusted OR	95% CI		*p*-value*
Abstainers	Ref			
Low risk drinkers (1–7)	0.95	0.66	1.38	0.785
High risk drinkers (8–19)	0.97	0.56	1.67	0.905
Hazardous drinkers (20+)	0.56	0.19	1.64	0.291
Recreational drug use				
No	Ref			
Yes	0.73	0.40	1.35	0.322
Do you do any vigorous intensity sport?				
No	Ref			
Yes	1.72	1.19	2.50	0.004
Do you do any moderate intensity sport?				
No	Ref			
Yes	1.42	1.03	1.95	0.031
When was the time you went to see a health personnel?				
Within the past six months	Ref			
More than six months but not more than a year ago	0.47	0.20	1.10	0.081
More than one year ago	1.60	0.61	4.22	0.338
Never	1.68	0.67	4.22	0.271
Where do you usually obtain health care?				
Public sector	Ref			
Private sector	1.06	0.69	1.63	0.776
In the past 12 months, have you been hospitalized for any illness?				
No	Ref			
Yes	0.44	0.30	0.63	0.000
HIV status awareness and treatment				
Not aware of HIV status	Ref			
Aware of HIV status and not on ARVs	1.18	0.87	1.60	0.286
Aware of HIV status and on ARVs	0.67	0.48	0.94	0.019

^a^Risk score based on a questionnaire for Alcohol Use Disorder Identification Test (AUDIT); *Significant at *p* < 0.05

### Multivariate logistic regression analysis

**Fig. 1:**
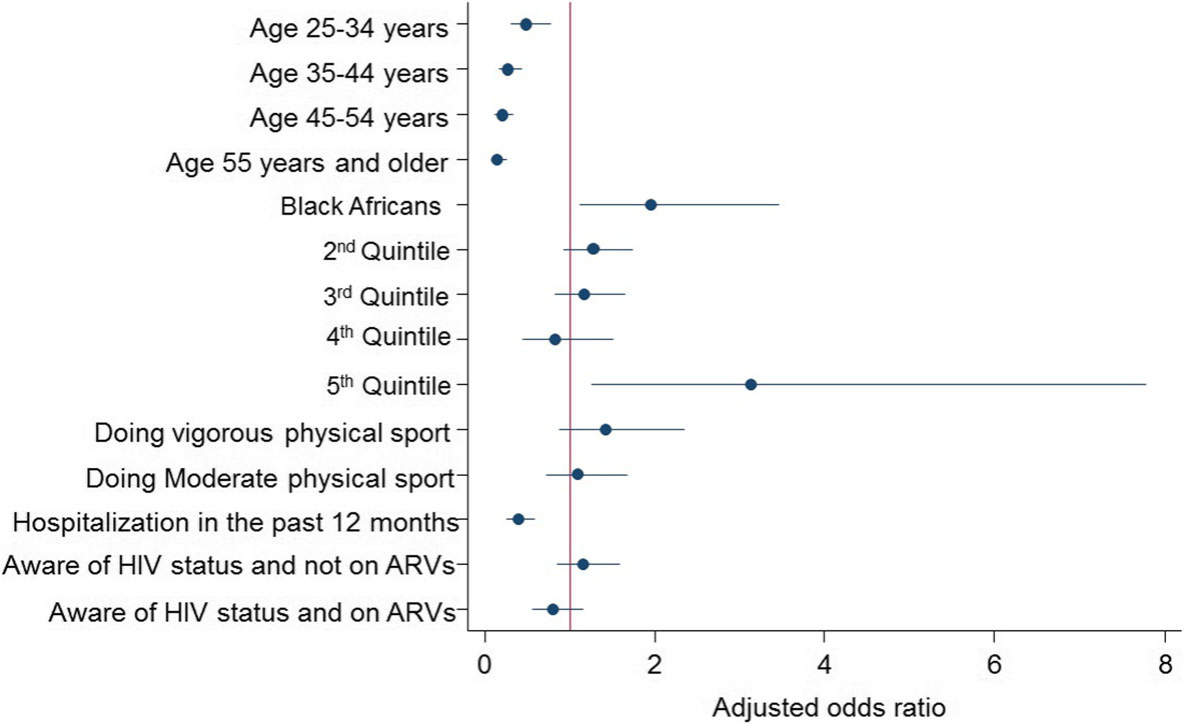
Adjusted model ORs and 95% confidence intervals significant at *p* < 0.05 for multivariate analysis of good/excellent self-rated health among HIV positive participants 15 years and older

Figure 1 shows final adjusted model for multivariate analysis of determinants of reporting good/excellent SRH among HIV positive participants 15 years and older. The effects and direction of the associations was similar to that observed in the bivariate analysis. However, only age, race, hospitalization in the last 12 months, and presence of chronic medical conditions remained significantly associated with reporting good/excellent SRH. The increased likelihood of reporting excellent/good SRH remained significantly associated with being Black African [OR = 1.97 (95%CI: 1.12–3.46), *p* = 0.019] and belonging to least poor households [OR = 3.13 (95%CI: 1.26–7.78), *p* = 0.014]. On the other hand the likelihood of reporting excellent/good SRH decreased with age and was significantly lower among those aged 25 to 34 years [OR = 0.49 (95% CI: 0.31–0.78), *p* = 0.003], 35 to 44 years [OR = 0.27 (95% CI: 0.17–0.44), *p* < 0.001], 45 to 54 years [OR = 0.20 (95% CI: 0.12–0.34), *p* < 0.001], and those 55 years and older [OR = 0.15 (95% CI: 0.09–0.26), p < 0.001] relative to those aged 15–24 years. In addition, the likelihood of reporting excellent/good SRH decreased significantly with reported hospitalization in the past 12 months [OR = 0.40 (95% CI: 0.26–0.60), *p* < 0.001].

## Discussion

The findings of this nationally representative study revealed that the majority of HIV infected South Africans 15 years and older considered their health as good/excellent. This might be attributable to a shift in the perception of HIV as fatal disease to a manageable condition due to advances in treatment and prevention [1–4]. Similar observations of improvements in self-reported health with changes in the HIV epidemic have also been made in other countries [12, 13]. Furthermore, the findings showed that the reporting of good/excellent SRH varied by demographic characteristics, life style and health-related factors. Similarly, differences in the distribution of SRH by socio-demographic, health and lifestyle conditions have been observed in other populations [5–10].

The increased likelihood of reporting good/excellent SRH among Black Africans compared to other race groups might be attributable to the fact that HIV has become increasingly ‘normalised’ in terms of social acceptance and acknowledgement [22]. Hence, the perceptions about the health implications of HIV have become more positive. On the other hand among minority race groups low social exposure, lack of acceptability and /or normalization of HIV informs underlying negative perceptions about the nature of the epidemic [23, 24], and may have negative implications among the HIV positive regarding perceptions about their health.

The results also showed that reporting of good/excellent SRH was associated with individuals from high SES households. Individual economic conditions are obviously a basic factor contributing to a good state of health. Evidence has shown that the higher the SES, the lower the prevalence and/or incidence of health problems, illness, disease and death [25]. Likewise other studies have also shown that the higher the SES the higher the better the self-evaluation of health status among HIV-infected individuals [13, 17, 18]. Is has also been postulated that lower SES individuals are more pessimistic in their health ratings because they have fewer material and social resources with which to deal with their conditions, leading to a higher level of suffering and poor self-rated health [26].

The observed SRH age gradient probably reflects worsening health conditions with older age [6, 7]. In older age health-related functional problems are associated with diminishing immune system and this may be exacerbated by HIV infection which also affects the immune system [14]. This indicates that those who are aging with HIV may be particularly more vulnerable to poor health. In South Africa, older adults make up a growing proportion of people living with HIV. This suggests that more attention should be given to the elderly leaving with HIV.

The decreased likelihood of reporting good/excellent SRH with reported hospitalization in the past 12 months is indicative of poor health. Other studies found that that people with poor SRH presented higher mean number of days in bed and average number of visits to the doctor in the previous 12 months [11, 27, 28]. Health complications in HIV infected individuals have been shown to be responsible for hospital admissions especially among those not initiated on treatment [29]. Others contend that in addition to HIV-related condition hospitalization could be either due to other comorbid conditions or a combination of both [30]. Further research is needed to investigate the determinants of poor health status and hospitalization among PLHIV already on ARVs.

## Limitations

One of the limitations of this study is the fact that the behavioural data are self-reported /increasing the likelihood of recall and social desirability bias leading to under reporting or over porting, respectively [31]. The cross sectional nature of the study can only demonstrate an association and precludes the establishment the causal mechanism that results in good/excellent SRH. Nevertheless, the strength of this analysis is the fact that it is based on a large nationally representative sample that can be used to draw inference about factors associated with good/excellent SRH among HIV infected individuals in the country.

## Conclusion

The findings suggest that given the increasing number of older adults leaving with HIV in the country there is a need for the development of appropriate social support services for the elderly to improve their health status. The findings also highlight the importance of addressing socio-economic inequalities for improved general health among PLHIV. Finally, SRH may constitute additional means of evaluating general health of PLHIV especially in the context of ARV treatment, where there’s a great expectation for people to live healthy lives with HIV. However, more research is needed to investigate the utility of self-rated and the broader health-related quality of life for HIV related care and management for PLHIV.

## Abbreviations

AGYW: Adolescent girls and young women
AIDS: Acquired immune deficiency syndrome
ARV: Antiretroviral
AUDIT: Alcohol Use Disorder Identification Test
CI: Confidence intervals
DBS: Dried blood spot
EAs: Enumeration areas (EAs)
EAs: Enumerator areas
HIV: Human immunodeficiency virus
MCA: Multiple correspondence analyses
OR: Odds ratios
PLHIV: People living with HIV
SES: Socio-economic status
SRH: Self rated health
USA: United States of America
